# ‘Getting involved in research’: a co-created, co-delivered and co-analysed course for those with lived experience of health and social care services

**DOI:** 10.1186/s40900-022-00353-x

**Published:** 2022-05-16

**Authors:** Carolyn Blair, Paul Best, Patricia Burns, Anne Campbell, Gavin Davidson, Joe Duffy, Anne Johnston, Berni Kelly, Campbell Killick, Denise Mac Dermott, Alan Maddock, Claire Jane McCartan, Paula McFadden, Anne McGlade, Lorna Montgomery, Sonia Patton, Dirk Schubotz, Brian Taylor, Fiona Templeton, Paul Webb, Chris White, Jade Yap

**Affiliations:** 1 School of Nursing and Midwifery, Queen’s University Belfast, Medical Biology Centre, 97 Lisburn Road, Belfast, BT9 7BL Northern Ireland; 2 School of Social Sciences, Education and Social Work, Queen’s University Belfast, 6 College Park, Belfast, Northern Ireland; 3 School of Applied Social & Policy Sciences, Shore Road, Ulster University, Northern Ireland; 4 Praxis Care, 25-31 Lisburn Road, Belfast, Northern Ireland; 5 Strategic Planning and Performance Group, Department of Health, 12-22 Linenhall Street, Belfast, Northern Ireland; 6 Trinity College Dublin, College Green, Dublin 2, Ireland; 7 Representative with Lived Experience of Health and Social Care Services, Belfast, Northern Ireland; 8 Mental Health Foundation, Colechurch House, London Bridge, London, Northern Ireland

**Keywords:** Participatory research, Health and social care education, Patient and public Involvement (PPI), Lived experience, Service users, Participatory theme elicitation (PTE)

## Abstract

**Background:**

‘Getting Involved in Research’ was co-created and delivered by a multi-organisational group to provide an accessible introduction to research for those with lived experience of health and social care services.

**Method:**

The evaluation of participants’ perceptions adopted an exploratory mixed method research design and aimed to gather data to provide an in-depth understanding of the participants’ experience of ‘Getting Involved in Research’ through the co-researchers’ analysis of qualitative data using Participatory Theme Elicitation (PTE). PTE was used with the qualitative data to promote co-analysis by the course development group; analyses from an independent academic was also used to further validate the method of PTE.

**Results:**

Thirty-five participants in total participated in ‘Getting Involved in Research’. Age ranges varied from 19 to 73 years old. Participants were predominately female (n = 24), five males participated (n = 5) and there was one participant who identified as non-binary (n = 1). Six core themes were identified using the PTE approach: (1) A Meaningful Participatory Approach (2) Increasing the Confidence of Participants (3) Interactive Online Format (4) An Ambient Learning Environment (5) A Desire for Future Courses (6) A Balance of Course Content and Discussion. Participants in ‘Getting Involved in Research’ reported that the content of the training was applicable, relevant, fostered awareness of research methods and anticipated that it would support their involvement in research.

**Conclusion:**

‘Getting Involved in Research’ has contributed innovatively to the evidence base for how to engage with and motivate those who have experience of health and social care to become actively involved in research. This study demonstrates that ‘Getting Involved in Research’ may be helpful to train those with lived experience and their care partners however, further research following up on the application of the course learning would be required to ascertain effectiveness.

**Future directions:**

Future research should explore methods to apply research skills in practice to further develop participants’ confidence in using the skills gained through ‘Getting Involved in Research’.

**Supplementary Information:**

The online version contains supplementary material available at 10.1186/s40900-022-00353-x.

## Plain English summary

The aim of this study was to gather information to help us understand the experience of participants’ undertaking a research course called ‘Getting Involved in Research’. A group of individuals including those with lived experience of health and social care services, academics, community and voluntary sector workers and a representative from the Department of Health in Northern Ireland worked together to develop a course to encourage and support people to engage with research. The ‘Getting Involved in Research’, course was designed to provide an understandable introduction to research for those people with lived experience of health and social care. We did this because research in health and social care sector should involve the patients and public who it is intended to help. The evaluation of the course had two distinct phases; we asked course participants to complete a survey before and after the course (pre- and post-course survey) and also asked them to complete a journal reflecting on their experiences after each lecture. This paper gives an overview of the profile of course participants and their responses to the survey questions. The survey answers were analysed using an approach to analysing information which encourages involvement from people with a range of experience of research methods, (known as Participatory Theme Elicitation). Thirty-five participants in total participated in ‘Getting Involved in Research’. Age ranges varied from 19 to 73 years old. Participants were predominately female (n = 24), five males participated (n = 5) and there was one participant who identified as non-binary (n = 1). Participants in ‘Getting Involved in Research’ reported that the content of the course was relevant, encouraged awareness of research methods and encourage their future involvement in research.

**Supplementary Information:**

The online version contains supplementary material available at 10.1186/s40900-022-00353-x.

## Background

In health and social care research, the research intended to benefit those with lived experience of health and social care services^1^ is traditionally remote in its design, delivery and analysis; however, there is a growing impetus to challenge this approach [1]. The National Institute for Health Research [2, p.11] state,

The suggestion that members of the public are ‘subjects’ or ‘silent partners’ in research is no longer a tenable position to maintain for any research organisation wishing to fund high quality research. Partnership, reciprocity and openness are now fundamental to how research is done and to the successful translation of research results into practice.

Involving those with lived experience in the research process represents a radical and much needed shift in how knowledge is produced within academic and healthcare organisations. Unlike conventional academic researchers, those with lived experience publicly and purposefully bring their ‘lived expertise’ to the work of research gained through experiences of contact with health and social care services [3]. The value of including those with lived experience in research and professional education is also widely recognised as contributing to good practice, while also challenging and transforming dominant biomedical ways of knowing and practicing [4, 5—Authors' own]. However, there are concerns that some forms of involvement of those with lived experience in research and education can be an exercise of tokenism [6–8]. When positioned as co-researchers, those with lived experience can be undervalued in research by conventional researchers given their lack of involvement in research methods training and, in some cases, this is augmented due to stigmatic experiences or personal doubts of their own capacity [9, 10]. Therefore, in order to move from tokenistic involvement to more authentic engagement; research training is necessary to support those with lived experience’s involvement in research [11–18]. Considering that public and patient involvement (PPI) in research is defined as “research being carried out ‘with’ or ‘by’ members of the public rather than ‘to’, ‘about’ or ‘for’ them.” [18, Pg. 6] it is essential to ensure that involvement in research is empowering rather than debilitating. Brett et al. [19, p.641] suggest,

Offering service user training in research methodology may help maximize the service user involvement and empower service users in their contributions to the design of the study, providing service users with the tools to discuss outcomes and formulate questions rather than limiting their involvement to accounts of their experiences.

Evidence suggests that training can help those with lived experience feel more confident, empowered and effective in their contributions [20–23—Authors' own]. Only a few research training programmes for people with lived experience exist that have been explicitly co-designed with those who have lived experience. As Blackburn et al., [24, p.1] state “The extent, quality and impact of PPI in primary care research is inconsistent across research design and topics. Pockets of good practice were identified making a positive impact on research.” Although training has been recommended for those with lived experience in research, little is known about exactly what training is needed and what has most impact.

In a valuable co-produced training programme, Stanley et al. [25] explore the nature of lived expertise and how best to share this knowledge to maximise the impact and benefit of involvement. Participants reported feeling more confident about involvement, they were clearer about their expectations and had increased understanding of how to use their lived experience and use it constructively. Stanley et al.’s [25] experience of developing and delivering this training programme has confirmed that there is potentially a gap in current training for patients and the public especially at the commencement of their involvement in research [25].

A mapping exercise conducted over fifteen months in 2002–03 to identify such training initiatives in health and social care, found just twenty-six initiatives across England, with twelve of these programmes providing training on the analysis and interpretation of data [26, 27]. It should be noted that this mapping exercise took place approximately twenty years ago, however findings are significant and the paucity of work in this area highlights an evident gap. The training programmes were varied in respect of design, length and content of provision, ranging from a single day duration to several months [27]. Findings from this study suggested that key aspects of successful training were centred on increasing confidence to contribute which was developed in a 'safe' mutually supportive environment [27]. Increasing research confidence was also the area of focus in Marshal et al.’s, [28] study which reports on introductory research skills training designed for people with mental illness to support improving their employment and productive activities of everyday living through the development of ‘a Clubhouse’ [29]. In this study Marshal et al.’s, [28] found increased confidence in performing roles relevant to research activities in Clubhouse settings however, there were no significant improvements in research self-efficacy related to more general research skills. Horobin et al., [30] took a more focused approach and piloted a lay assessors training programme and their findings suggest that the sharing of varied experiences and knowledge and the ‘learn by doing’ approach was particularly valued. Similarly, Richardson et al. [1] reports on a co-produced two-day research awareness training programme for mental health service users and carers. Public involvement included thirteen participants in the training and three participants in the evaluation team. The evaluation found that participants gained greater confidence in their ability to volunteer to get involved but also highlighted the difficulties of meeting the training needs of a diverse group with varying experiences and expectations. In particular, Richardson et al. [1] found that public involvement in the analysis and interpretation stages, was fulfilling for the participants and increased the authenticity of the evaluation findings. However, despite these findings being useful there is a paucity of recent studies gauging what content and quantity of training would be useful for those with lived experience who have little or no familiarity in conducting research.

In response, in Northern Ireland (NI), a collaboration of service-users and carers with lived experience of health and social care services; representatives from the Department of Health (DoH), the Mental Health Foundation (MHF), Praxis Care, Queen’s University Belfast (QUB) and Ulster University (UU) formed to design a course which would facilitate people with lived experience to engage with and conduct research. This work was funded through the Disability Research on Independent Living and Learning (DRILL) programme which ensured that everyone’s involvement, if not already funded, was paid. The course was named ‘Getting Involved in Research’ and designed to provide an accessible introduction to research for those with little or no previous experience. The team met on a monthly basis over the course of a year during the pandemic, to discuss and plan ten, two-hour lectures which were delivered over one week. The course aimed to develop participants’ knowledge of conducting research and its application in health and social care, while also considering the complexities of the role of lived experience in the research process. An additional goal was to develop participants’ awareness of the opportunities to further develop their involvement in research. The ‘Getting Involved in Research’ course development group embedded a participatory approach in its design, delivery and evaluation. Participants in the course were encouraged to recognise themselves as stakeholders in the project with the assurance that their collective perspectives would contribute to future course design [31—Author’s own].

It is often the case in co-production and co-design that stakeholders and partners from outside of university settings are not included in the data analysis despite the evidence that participatory data analysis has the potential to transform practice and strengthen the methodological rigour of results [32–35]. Involving stakeholders as co-researchers in the data analysis process can enrich the process by creating more relevant, meaningful, and valid results [35, 36—Authors' own], [37, 38]. In response our team aimed to enhance our collective consciousness through using co-production in all aspects of the research process [31—Author’s own]. To ensure methodological rigour and full involvement, Best et al., [36—Author’s own] suggests using Participatory Theme Elicitation (PTE) as an accessible way to include co-researchers in qualitative data analysis. The course development group agreed to use PTE to help address the limitations of participatory analysis by democratising the process with a representative sample of the course development group. PTE is a five-step process which provides an accessible and understandable process for co-researchers and academic researchers [23, 36, 39–41—Authors' own]. As a relatively new approach first developed in 2017, PTE has shown promise in previous mental health research with youth from schools [36—Author’s own], co-researchers from the public to examine group-based video-conferencing for adults with depression [40—Author’s own], with co-researchers to examine an exercise intervention for people with serious mental illness [23—Author’s own] and in engaging teachers and school leaders in analysis of findings from a school-based mental health intervention [41—Author’s own]. Findings support PTE as an effective, accessible and valid method to use with lay-researchers [23, 36, 39–41—All author’s own], but this has yet to be tested using an online methodology with academics, community and voluntary sector partners and those with lived experience.

This paper will add to the evidence base for course designs to engage with and encourage those who have experience of health and social care to become actively involved in research. Furthermore, this paper will examine the use of PTE using the participants’ qualitative data analysed by a representative group of stakeholders from the course development group of ‘Getting Involved in Research’.

## Research design

The methods described within this article focus on the recruitment and data collection for ‘Getting Involved in Research’ and a description of the method for PTE analysis is described in the analysis section.

### Aim and objectives

The aim of this study was to gather data to provide an in-depth understanding of the participants’ experience of ‘Getting Involved in Research’ through the co-researchers’ analysis of qualitative data using PTE.

The objectives included:

1. To investigate whether participants perceived the course as engaging
2. To explore where participants perceived the course as participatory
3. To assess what could be improved to make it ‘Getting Involved in Research’ more useful for future course participants

### Study context

The study adopted an exploratory mixed method research design which is recommended in the early development stages of course design [42]. This flexible path of inquiry provided rich information to help establish an in-depth understanding of participants’ experience and provided useful information for course development. This study adopted a pre- and post-course survey and reflective journals to explore the experiences of participants in ‘Getting Involved in Research’. Given the current circumstances (Covid-19) the course was delivered online, and all data collection was also conducted online using Microsoft Forms (MS forms).

### Design

In the pre-course survey, we asked open-ended questions relating to the participants’ prior experiences and aspirations regarding their involvement of ‘Getting Involved in Research’. In the post-course survey, the aim was to ascertain whether participants perceived the course as engaging while also assessing the relevance and potential for the practical application of the course. The reflective journal was designed to be completed after each lecture and focused on participants’ experience of the lectures (Did the lecture effectively engage your attention? How relevant did you feel the lecture was? How do you feel the lecture affected your confidence? How satisfying did you feel the lecture was?) to ascertain which parts of the course were well received and which were more difficult for participants to understand which will aid in future course design. The research design was framed using Keller’s *ARCS model of motivation design* [43–45], focusing on how effectively the participants deemed the lectures to engage their attention, how relevant they found them, how it affected their confidence, and how satisfied they were with the lectures. For the purpose of this publication, we will focus on a descriptive overview of the profile of participants and qualitative data gained through their response to the open-ended survey questions. The survey questions were developed and designed collaboratively with academic researchers, community and voluntary sector partners, those with lived experience and a DoH representative.

### Procedure

All participants were informed about the study via an invitation email that provided details of the study involving pre-course survey, post-course survey, reflective journals, a participant information sheet, and a consent form. The consent form, pre-course survey, post-course survey and reflective journals were accessed online via MS forms links. Consenting participants were encouraged to complete their reflective journal after the lecture and the post-course survey after the final lecture. All participants were provided with a MS form link for the reflective journal via email each day and after each lecture participants were encouraged to complete their reflective journal via the MS Teams chat function using a reminder of the MS forms link. Participants were advised to contact the course lead if they were experiencing any difficulties or required any support in relation to the course and advised to contact the research lead if they experienced any difficulties with MS forms or had any queries in regards to the evaluation.

### Participants and sampling

The programme was open to participants who had lived experience of health and social care, carers of those with lived experience and professionals in related fields. Flyers advertising the course were disseminated widely via community and voluntary sector organisations and advertisement in the Northern Ireland Health and Social Care Board newsletter.

### Ethical consideration

Ethical approval was obtained from the Research Ethics Committee of School of Social Sciences Education and Social Work at Queen’s University Belfast prior to study commencement. All participants were informed about the study via an invitation email that provided details of the study involving pre-course survey, post-course survey, reflective journals, a participant information sheet, and a consent form. All participants provided informed consent.

### Data analysis and interpretation

A convenience sample approach [46] was used to recruit the co-researchers from the Course Development Group. Recruitment was achieved through an invitation email. Eight course development group members were recruited for the PTE analysis process. Among the four males and four females were two academics, one DoH representative, three community and voluntary sector representatives and two participants with lived experience. PTE was conducted through an accessible, five step approach outlined by Best et al. [39—Author’s own], (1) data selection, (2) capacity building, (3) data sorting, (4) data grouping and (5) data analysis and interpretation. The PTE method required co-researchers’ involvement in Steps two, three and five. In addition to the PTE participants, one academic researcher and one co-researcher with lived experience were responsible for steps one and four and facilitated the final data analysis and interpretation session in step five.

1. Data Selection

In step one, the academic researcher and co-researcher with lived experience independently read all of the anonymised participant post-course surveys and reflective journal data and selected quotes that that could be easily understood as ‘standalone statements’. No formal coding of data took place during this activity and no conscious effort was made to group data together or select quotes based on potential significance. The only terms of reference were that the quotes were broadly representative of the larger dataset. The academic researcher and researcher with lived experience agreed on the identified quotes that were the same and discussed the quotations which differed in a two-hour meeting. All statements were re-checked before being presented to the co-researchers.

2. Capacity Building

**Fig. 1:**
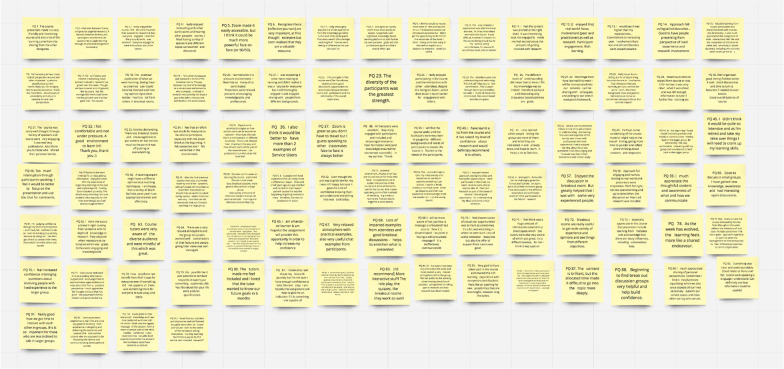
Image of Miro Board containing post-it notes with respondents’ quotes

In step two, the academic researcher provided each of the eight co-researchers with individual Miro boards (see Fig. 1: Example Miro board) via email which included all 94 quotes (Additional file 1). Miro is an online collaborative whiteboarding platform designed to enable teams to work effectively together, from brainstorming with digital sticky notes. A twenty-minute training session via Zoom was provided which included an overview of the PTE process and instructions on using Miro and sorting data. The aim was to equip the co-researchers to complete the task on their individualised Miro board and help guide decision making during the analysis and interpretation step. The co-researchers were advised to initially spend time reading the ‘sticky notes’ (the participant quotes) individually to familiarise themselves with the data and guided to categorise their initial codes based on what they deemed important to those who participated in ‘Getting Involved in Research’. They were then asked to sort the quotes into groups based on what they thought was the most apparent themes in the data. No further instructions were given by the research team as we did not want to influence the analysis of the data or obstruct the emergence of unique perspectives from the co-researchers. During the training, the academic researcher ensured that all co-researchers were aware that she was available to answer any questions on the PTE process. This session was recorded and made available for any participants who requested to watch it again.

3. Data Sorting

**Fig. 2:**
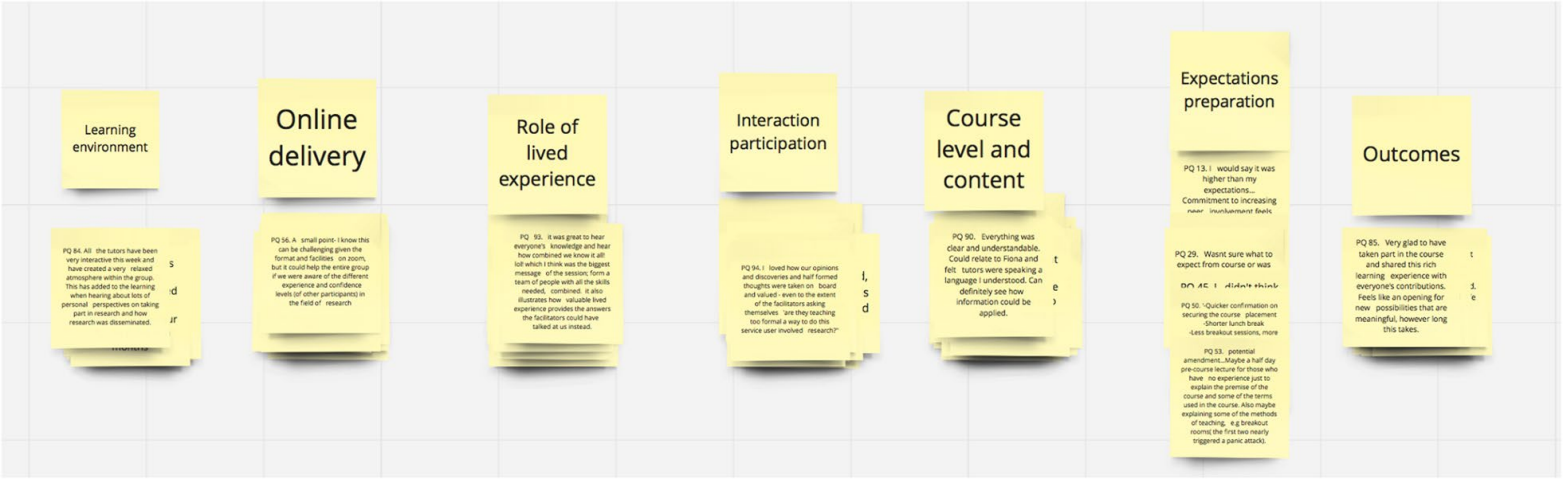
Example of a Co-Researcher’s Miro board after completion of sorting task

In step three, the 94 quotes were given to the co-researchers, with each (n = 8) receiving their Miro board with post-it notes individually labelled 1–94. These co-researchers sorted the quotes independently and were given five days to complete the task, advising it should take approximately one hour to complete. After completion of the sorting process, each co-researcher had between four and twelve groups for the quotes, which were labelled and re-ordered in piles (Fig. 2: Example of a Co-Researcher’s Board).

4. Data Grouping

**Fig. 3:**
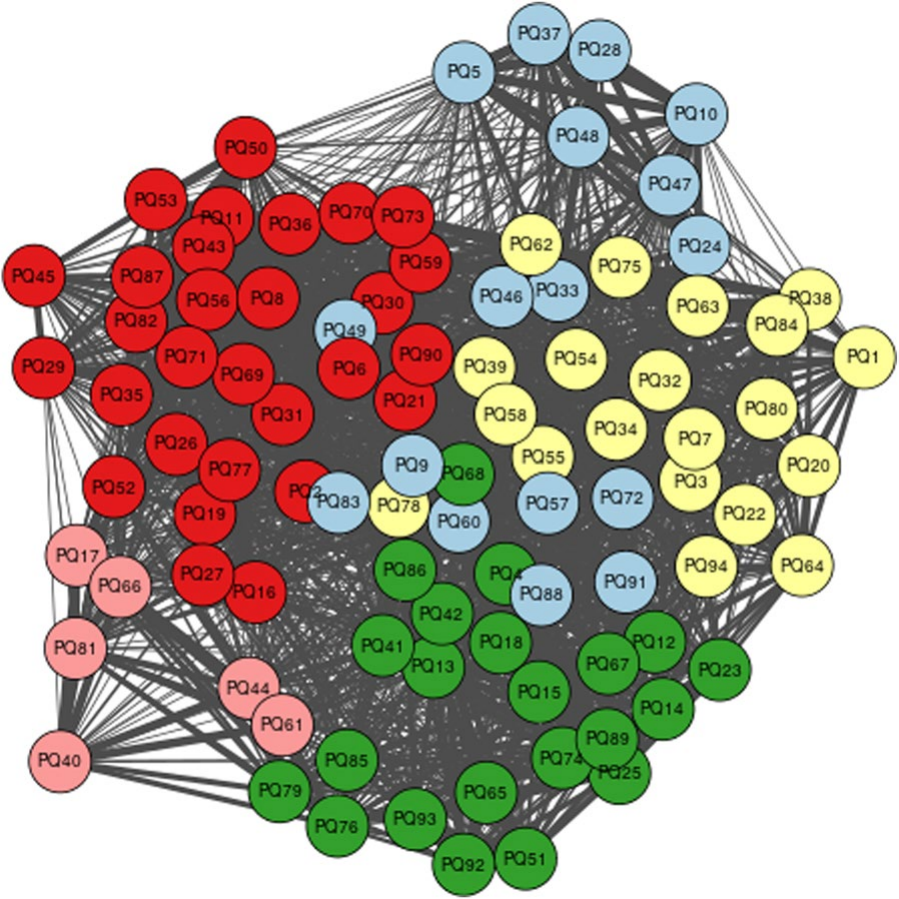
Network analysis

The objective of step four was to construct a set of groups of quotes that reflect the general consensus between the co-researchers’ themes identified in step three: Data Sorting. The themes organised by each co-researcher were assigned a number and inputted into a Microsoft Excel spreadsheet. Three columns were utilised to distinguish (1) Topic (Excerpt ID), (2) Person (Co-researcher ID) and (3) Group. For example, if co-researcher 1 grouped quotes 3, 8 and 37 together into the same theme, then this would be assigned a group ID for example, Grp1. This process was complete when all quotes had been labelled into numerical groups. The excel file was uploaded to online software created by Queen’s University Belfast [36—Author’s own] for network analysis. The analysis creates a network of quotes, allocating edge weights from the number of researchers who paired the quotes in the same theme [36—Author’s own]. The participants individual analyses are combined by applying a community detection algorithm to the weighted network which ensures that the most common combinations in the individual analyses are kept together [47]. The network of quotes produced five core groupings (Fig. 3: Network Analysis) with the thickness of the edge indicative of the number of co-researchers who sorted the pair of quotes into the same group. The colour indicates groups identified in step 3: Group 1 (red), Group 2 (green), Group 3 (light yellow) and Group 4 (light pink) and Group five (light blue). The different colours in the diagrams represent the different groupings found by the algorithm. The group of co-researchers were informed that the information regarding the strength of relationship between groups and quotes could be understood visually from the network diagrams by looking at the proximity of the nodes to each other and the thickness of lines connecting them together.

5. Data Analysis and Interpretation

**Fig. 4:**
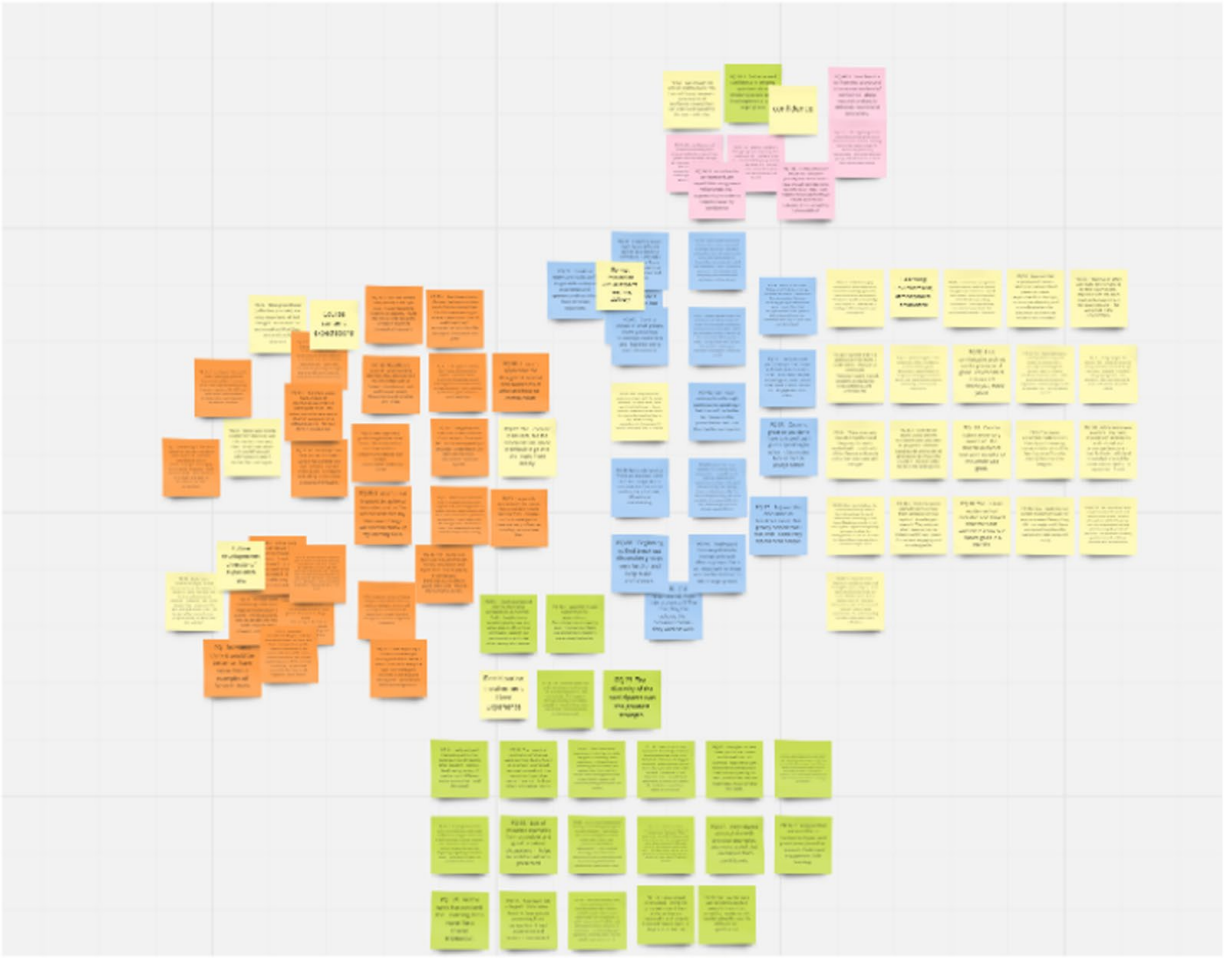
Miro board post co-research group discussion

One week after the data sorting took place, the network diagram along with the quotes within each group was presented to the co-researchers via Zoom. All eight co-researchers attended. The co-researchers were given access to a communal Miro board which was colour-coded to reflect the Network analysis prior to the meeting. The network analysis diagram and corresponding Miro board from step four was used to help encourage discussion on the identified themes between the academic research, co-research with lived experience and the co-researchers. Informed by the principles of Braun and Clarke’s [48, 49] thematic analysis, processes were then used to explore the groups from the network analysis and interpret possible themes. The discussion regarding the labelling of themes and reassignment of quotes to different groups was recorded during the session (See Fig. 4: Miro board post co-research group discussion). Using the network analysis and discussion, the co-researchers agreed to create an additional group. The reassignment of quotes into other themes was expected as this is part of the iterative and collaborative process of PTE analysis and co-production.

### Validation of PTE methodology

Given that further work is required to validate the method of PTE [23, 36, 39–41—All author’s own]; we also compared analyses from an independent academic, who was not involved in the course development group, delivery of the course material or PTE workshops. This person was given the full excel data set to read, then asked to independently and thematically group the 94 extracted quotes. This academic was not given the titles of the themes, however she was aware of the aims and objectives of the course and had access to the questions which were asked in the pre and post course survey and the reflective journal. The only guidance given to was that the quotes for the participant data set were divided into six core themes. Post this exercise, discussions took place regarding the co-researchers’ categorical groups wherein similarities and disagreements were noted.

## Results

### Overview of participant profile

Thirty-five participants in total participated in ‘Getting Involved in Research’. One week prior to the commencement of the course, all registered participants were invited to be involved in the research study and informed that participation would be voluntary and non-obligatory to attending the course. Thirty participants consented to participate in the evaluation of the course including Statutory Sector Professionals (n = 13), C&V Sector Professionals (n = 6), those with lived experience of health and social care (n = 7) and carers with lived experience (n = 4). Age ranges varied from 19 to 73 years old. Participants were predominately female (n = 24), five males participated (n = 5) and there was one participant who identified as non-binary (n = 1). In regard to academic background, nine participants had completed postgraduate degrees (n = 9), fourteen participants had completed an undergraduate degree (n = 14), three participants stated their academic background was diploma level (n = 2), two participants stated GCSEs (n = 2) and two participants stated that they are currently completing an undergraduate degree (n = 2). Twenty-one (n = 21) participants responded to the post-course survey. Two participants dropped out of the course stating the reason of unprecedented work commitments.

### Thematic analysis using PTE

The six core themes that were identified by the co-researchers through the PTE approach were as follows: (1) A Meaningful Participatory Approach (2) Increasing the Confidence of Participants (3) Interactive Online Format (4) An Ambient Learning Environment (5) A Desire for Future Courses (6) A Balance of Course Content and Discussion.

### Validation of PTE methodology

Having read the entire data set and being independent from the course development of ‘Getting Involved in Research’, overall, the academic researcher thematically grouped 78% of quotes in the same grouping as the co-researchers. 90% of quotes in the same grouping as the co-researchers’ label of Participatory Approach. 75% of quotes in the same grouping as the co-researchers’ label of Confidence. 94% of quotes in the same grouping as the co-researchers’ label of Format. 85% of quotes in the same grouping as the co-researchers’ label of Learning Environment. 33% of quotes in the same grouping as the co-researchers’ label of Future Developments. 89% of quotes in the same grouping as the co-researchers label of Course Content (See Additional file 2).

For the large majority of quotes there was consensus through independent adjudication, despite minor differences in interpretation of quotes. Overall, there was strong similarity in categorical groupings. Discussions post this exercise found that although the future developments category was significantly lower agreement (33% similarity), the independent researcher categorised the co-researcher sub-theme of diversity of experience as a core theme as opposed to being in the same theme as Future Developments. There was minor disagreement between what was deemed to be Course Content (89% similarity) and Format (94% similarity). Correspondingly, a minority of quotes which the co-researchers deemed to be categorically learning environment was categorised as Course Content (85% similarity). A minority of quotes (75%) which were deemed to relate to confidence by the co-researchers was categorised as either course content or participatory approach. There was strong agreement in the theme of participatory approach (90% similarity) with only one quote which differed and was categorised in the same theme as future developments.

1. A Meaningful Participatory Approach

The co-researchers identified that including those with lived experience as stakeholders in the research design and delivery of ‘Getting Involved in Research’ meaningfully improved the ‘service-user‐centredness’ of the design. This theme had two subthemes: (a) the benefit of involvement (b) the contributions of lived experience. The first sub-theme related mostly to the positive impact involvement can have on individuals such as the sense of non-tokenistic involvement. The second sub-theme related the unique contributions that a participatory approach can bring to the research process.

“[The] approach felt collegial/collaborative. Interesting throughout. Presenters had prepared up to date, rich, thoughtful content and participative exercises. Valuable learning from course participants was shared/received with interest, not defensively. Good to have people presenting from perspective of lived experience and research involvement.” [ID: PQ14]

Data indicated that the participatory approach of ‘Getting involved in Research’ was deemed to have the potential to make a very positive impact on society. One participant described their experience of the course in reflection of their journey contributing as expert through experience.

“It is people with lived experience that can really make a difference, I began three and a half years ago as a PPI member and trained on their 'expert patient' course this was the beginning of getting involved in many initiatives, this was co-production in action” [ID: PQ 51]

As trust and confidence increased, the impact of lived experience was noted to move from an abstract construct to more real as the week progressed. Authenticity in course tutors and other participants was valued highly, with one participant stating:

“I would say it was higher than my expectations... Commitment to increasing peer involvement feels real and all contributions were valued/valuable.” [ID: PQ 13]

Another dimension which came through when considering involvement was the uncomfortable labelling of ‘service users’ and the sensitivity required when involving those with lived experience as not wholly identifiable by their service user status.

“I much appreciated sharing of personal perspectives. Sometimes I find it helpful (more equalising) when we also value aspects of our lives sensitively out with our service status and roles when we say who we are.” [ID: PQ 89].

Similarly, the qualitative data largely confirmed that participants felt valued for their lived experience and potential contributions. As a participant noted, “Yes you felt like it was possible to conduct research, it wasn't just something academics did. You felt valued for your life skills and not qualifications.” [ID: PQ 79].

2. Increasing the Confidence of Participants

Preliminary findings suggest that ‘Getting Involved in Research’ has increased the majority of participants’ confidence in conducting research. One participant noted that gaining knowledge, confidence and subsequent desire to recommend the course were linked: “I have learnt a lot from the course, and it has raised my level of confidence about research and would definitely recommend it to others. [ID: PQ 40].”

Participation, collaboration with other participants and active involvement in the course were also related to confidence, as reflected by one participant:

“I'm judging confidence through my level of participation - and I have felt confident to do so - even to initiate group activity because by doing so I can then get others to convey their views, from which I and the others can benefit.” [ID: PQ 61.]

Ated into the structure.

The theme of increasing confidence and participation was also clearly linked to time for discussion in small breakout rooms being integrated into the structure, this was notably appreciated by the majority of participants. As a participant stated,

“At the beginning I found myself interacting within small breakout rooms but not feeding back in the bigger group. By Wednesday I felt very comfortable interacting with my group and volunteered to feed back to the bigger group.” [ID: PQ 44]

The structure of the course seemed manageable to the large majority of participants despite the course being intensive (ten two hour lectures). This was also an important component related to increasing the confidence of participants. A participant felt that the way in which the format and content was organised helped to increase the confidence of participants,

“My confidence and interest in becoming more actively involved in research has grown over this week. Though we have covered a lot of ground, the structure has felt manageable, and I could sense the thought and care that has gone into this course.” [ID: PQ 17]

3. Interactive Online Format

This theme had two subthemes (a) Interaction (b) Virtual aspects teaching and delivery. The first sub-theme related mostly to the positive impact interactive elements within the course had on participants’ enjoyment of and engagement with the course. The second sub-theme related to the fact that the course was delivered online as opposed to face-to face. Generally, the participants were contented with the structure and organisation of the course. As a participant described,

“Thanks to the team involved for an educational, informative, enjoyable week.

With my experience of organizing meetings in the past and organising and hosting Zoom sessions currently. The emails every morning I am sure avoided a few requests for the links even though you had already supplied them!” [ID: PQ 47]

Small groups were viewed positively and linked to improving the interactivity of the experience which also was a theme which overlapped with building confidence and increasing openness for participants. An example of the comments included “Good to discuss in small groups. I have gained new knowledge, awareness and had interesting open discussions” [ID: PQ 60.]

Similarly, this seemed to be linked to the ability to share and appreciate other participants experiences.

“Breakout rooms are really useful to get wide variety of experience and opinions and see things from different objectives” [ID: PQ 72]

“Really good how we got time to interact with each other in groups, this is so important for those who are less inclined to talk in larger groups.” [ID: PQ 91]

Participants also offered some helpful critique regarding the method by which the course was delivered being exclusively delivered remotely via Zoom. Limitations included the inability to interact with other participants as freely as face-face courses, however, there was an understanding that the restrictions surrounding Covid-19 dictated these circumstances. An example of related quotations include:

“Zoom made it easily accessible, but I think it could be much more powerful face-to-face (or 50/50).” [ID: PQ 5]

“I really enjoyed participating in the course and the interactions with other attendees, despite this being on Zoom...which does make it much harder for engagement with others.” [ID: PQ 24]

Despite the limitations of Zoom, the general consensus was that it was used in a way which engaged participants’ attention with a variety of methods which increased its effectiveness. As a participant described, “A teaching expert might have a different opinion about teaching techniques. I am happy that a variety of Zoom facilities were used in an appropriate manner and effectively.” [ID: PQ 48].

4. An Ambient Learning Environment

This theme was based on the learning environment created by the tutors delivering the course and significantly pointed to the appreciation of what seemed to be reflective practice. This core theme had one subtheme (1) atmosphere and ambience. This subtheme was directly linked to the participants’ ability to relax and engage with the experience despite the awareness of the diversity of the experience level of the group.

The learning environment was directly linked to the welcoming of feedback, questions and thoughts during the delivery of the course and the democratisation of the learning process. As one participant noted:

“I loved how our opinions and discoveries and half formed thoughts were taken on board and valued - even to the extent of the facilitators asking themselves 'are they teaching too formal a way to do this service user involved research?” [ID: PQ 94]

Authenticity again overlapped with the creation of a conducive learning environment, namely due to the skill of the tutors in their responsiveness and through applying the learning using contemporary examples.

“Approach felt engaging and human, relating to real world and presenters were responsive. Pitch felt right, not too overwhelming and up-to-date (when the discussion on Peer-Led research was included)” [ID: PQ 58]

A large majority of the participants commented on the value of a relaxed environment contributing to the atmosphere and ambience of the course. As one participant noted, “There was a very relaxed atmosphere and the group discussion worked well- combination of chat feature and people giving their views was well-managed.” [ID: PQ 64].

The atmosphere specifically contributed to calming anxieties and alleviating pressure especially for participants who had less experience of research. A participant described their appreciation of this, “I felt comfortable and not under pressure. A good environment to learn in!! Thank you, thank you:)” [ID: PQ 32].

5. A Desire for Future Courses

Participants strongly advised that they would like to see more courses such as this for those with lived experience or those working in related fields. They did, however, note that incorporating more discussion to displace some of the material could have been useful in contributing to more reflective learning. There was one subtheme of (1) diversity of experience alongside the core theme. This sub-theme was focused on their appreciation of the meaningful contributions of all participating in the course and the need to include more tutors with lived experience in future course designs.

The participants generally valued the balance of the course material including opportunities for further involvement in the course and also the opportunity to apply learning through an assessed assignment. This was deemed by the co-researcher to be an essential point for learning in future course development. One participant stated their perception of the most helpful parts of the course.

“A balance between theory and practice (applied research); a balance between delivery and participant involvement; an opportunity to apply learning through an assessed assignment (voluntary)” [ID: PQ 2]

One participant summarised the potential value of the condensing the material to aid in more interaction and engagement with participants. As a participant commented: “Perhaps some condensing of the course material might help in the overall timing, giving more time to ponder and reflect when thinking about answers and responses.” [ID: PQ 43].

Generally, participants stated that they very much appreciated the contributions of those with lived experience, however encouraged increased diversity of experience and more active involvement of those with lived experience for future course designs. As one participant noted “I also think it would be better to have more than 2 examples of Service Users” [ID: PQ 36].

Furthermore, there was a desire for more explicit explanation of how those with lived experience can be actively involved in a research team and how their contributions are made meaningful. As one participant queried:

“Maybe some activities/strategies on how service users can be involved in research - what does this look like in a real example, in different situations? The course showed how important this was, and how service users can be part of the research team (co-production), but what does this look like?” [ID: PQ 35]

Despite a little confusion over the pre-requisites for attendance and course content the participants generally noted that this was more of a strength than a limitation of the course. As one participant stated,

“I was expecting it to be more relating to nursing and didn't realise it was a course for everyone, but I still thoroughly enjoyed it and enjoyed mixing with people from different backgrounds.” [ID: PQ 21]

6. A Balance of Course Content and Discussion

The course development group deemed the ‘course content’ to be a significant theme as generally participants were satisfied with what was delivered on the course and the tutors achieved a balance of course content while incorporating time for discussion. However, a repeated theme was based around requiring more time for further explanation and discussion. Participants did note an understanding of the difficulties in matching the level of the course to all participants’ levels of research experience and knowledge. One sub-theme from this was (1) Expectations which included comments on the course being aligned with participants expectations of what they anticipated. However, there was notable consensus that although participants thought the diversity of participants did not detract from their satisfaction of the course, it was somewhat unexpected. As one participant noted, there was evidence of significant planning in the course content and again a distinctive appreciation of the value of tutors speaking from a position of having lived experience.

“The course was really well thought through. Variety of speakers and topics were very engaging. It seemed very professional. Also thank you to those who shared their personal stories.” [ID: PQ 31].

Again, the time allocated to cover the level of content may require consideration for future course planning. As a participant noted, “The content is brilliant, but the allocated time made it difficult to go into the topic more deeply.” [ID: PQ 87].

The diversity of the participants was an area which was oftentimes linked to course expectations and may have detracted from maximum interaction. However, as described by a participant, the discussion-based activities and small groups were an aid to increasing understanding, “The different levels of understanding did mean that at times I felt my knowledge was so limited therefore sat back and listened quietly. Discussion based activities are great [ID: PQ 26].”

An area which was raised consistently as a notable commendation of the course was the opportunities for future learning and engagement in research. As one participant noted, “I feel dissemination of actual real opportunities was brilliant as sometimes it's a bit overwhelming in where to even start. Lots of useful websites/ resources but also the offer of support from tutors was great” [ID: PQ 70].

Overall, the large majority of participants agreed that the course met and, in many cases, exceeded their expectations. As a participant explained:

“I said agree as I partly didn't know what exactly to expect. I expected a well organised, knowledge-based learning platform to both further my own personal goals and also professional goals and that's exactly what I got.” [ID: PQ 8]

## Discussion

### How engaging did course participants find ‘Getting Involved in Research’?

It is evident that the provision of appropriate training to equip those with lived experience with the necessary knowledge and skills is an essential requisite for involvement in any aspect of the research process [19, 25, 26, 50, 51] and is increasingly recommended by key organisations [11–18]. However, given the lack of research on this area, it is difficult to ascertain exactly which components make a successful course for this cohort of individuals. From the findings of ‘Getting Involved in Research’ we suggest that the motivation to attend and the retention of participants was a significant sign of the demand for such courses and of its success. Findings suggest that all of the participants who responded to the post-course survey enjoyed their experience of ‘Getting Involved in Research’ noting with particular reference the coherence of the PowerPoint presentations, the tutors’ delivery and the opportunity to contribute with an atmosphere which encouraged active participation. Participants perceived social interaction with learning as a very important factor which underlines the importance of the social aspects of the delivery of ‘Getting Involved in Research’. The findings showed that participants particularly enjoyed working in groups, in particular the discussions which correlates with previous findings of research courses for those with lived experience [27]. The small group settings provided opportunities for participants to share experiences, gain confidence and share information with others which enhanced the participative learning experience. This supports INVOLVE’s suggestion that training for those with lived experience should encompass a learner-centred approach, with participants taking an active role in their learning offering opportunities for interaction and sharing of participants’ experiences [18, 21, 25, 27]. Therefore, being able to apply a range of didactic, small group and interactive approaches to training delivery was essential and successfully achieved by the tutors delivering the training.

#### Did course participants find ‘Getting Involved in Research’ participatory?

Given that the majority of the participants commented positively on the participative approach to ‘Getting Involved in Research’, this indicates that the course successfully achieved its aim. The participants did comment that the learning environment promoted an atmosphere of power-sharing and democracy which was welcomed. Given the diverse nature of the course participants, the relaxed environment cultivated by the course tutors seemed to allay anxiety and helped participants to find the confidence to ask questions or/and share their personal experiences. Facilitation skills and the competencies of the tutors delivering the training were important to achieving an authentically participatory approach. The demonstration of knowledge and expertise in the training subject areas was also deemed to be a key component of the success of ‘Getting Involved in Research’. However, in particular, strong reference was made to the value of hearing more about the expert knowledge of tutors who had lived experience. As such, participants suggested that there is a need for more representation for those with lived experience in the delivery of the course material of ‘Getting Involved in Research’. Increase in user narratives, and service user involvement generally in education, have been found to facilitate students’ communication, partnership and advocacy skills [52—Author’s own]. Therefore, it is essential in future developments to action more creative ways to include those with lived experience in courses such as ‘Getting Involved in Research’ and potentially theorise the development and use of service-user knowledge [53]. Although ‘Getting Involved in Research’ brought a unique perspective through involving those with lived experience in the design and delivery of the course there is a continuing need to increase meaningful involvement. As Duffy et al. [52—Author’s own] states this is not merely a micro issue of improving individual practice, but also “a macro issue challenging the grounds on which groups become ‘othered’ and where notions of ‘deserving’ and ‘undeserving’ are constructed”. This also related to the area which was noted by course participants of labelling and identity being constructed based on ‘service user’ status alone. This was an area which findings confirm was handled with notable sensitivity and was well received by participants, however, more work in this area for future course design could be beneficial.

### What could be improved to make it ‘Getting Involved in Research’ more useful for future course participants?

‘Getting Involved in Research’ has successfully demonstrated a course which has been participatory in conception, delivery and evaluation. Given that wider concerns have been expressed about involving those with lived experience as an exercise of tokenism [6–8] it is notable that the majority of participants deemed the participatory approach in ‘Getting Involved in Research’ as authentic. Despite the positive findings of this project, to improve ‘Getting Involved in Research’ more concrete examples of participatory research endeavours would be well received plus a wider representation of those with lived experience delivering on the course. Given that the majority of participants stated that the course increased their confidence to contribute developed in a 'safe' environment, this correlates with previous findings of research courses for those with lived experience [27]. However, although the course did contain a written assessment which was favourably received and opportunities for further study and support were made available, we do not know whether participants’ research skills in practice have increased. Marshall et al.’s study [28] reported similar findings indicating that that provision of more extensive training may lead to more consistent changes in confidence. In particular, considering that Horobin et al.’s, [30] ‘learn by doing’ approach was particularly valued it may be worthwhile to integrate more practical research tasks into the ‘Getting Involved in Research’ training in order to increase research skills in practice. Furthermore, given that research work experience is known to increase research confidence [18], this may be an opportunity for future consideration in a follow-up course for those who participated in ‘Getting Involved in Research’. Generally, participants viewed that the structuring of the lectures as accessible combined with relevant and informative content. However, given that there was mixed feedback on whether the diversity of the group aided or hindered learning, this highlights the need for further work on how training can be more individualised or how experienced individuals can be meaningfully integrated into a group of people with mixed ability. Furthermore, given that some participants stated that they felt under-prepared and lacking in experience compared to other members of the group it is notable that this may have reduced their capacity and confidence to contribute. These findings correlate with Richardson et al.’s [1] study which also highlighted the difficulties of meeting the training needs of a diverse group with varying experiences and expectations. Therefore, more consideration of the potential struggles of those who were less experienced in the group through a screening process of applicants may also help to eliminate any power-imbalances and contribute to the parity of learning for future participants.

### Strengths, limitations and acceptability of PTE

From these findings we would suggest that PTE has proven reliability and acceptability as a participatory method of thematic analysis. Given that one individual with lived experience and the other coming from an academic research perspective selected the 94 quotes collaboratively, the team made efforts to ensure a fully participatory approach at every stage of the process. Furthermore, given that these researchers were not involved in the steps three and four of the PTE process and made efforts to remain impartial while facilitating step five, the potential for selection bias was minimised. The same eight co-researchers were present for each stage of the PTE process which permitted consistency during the process. Incorporating co-researchers with mixed backgrounds from the course development group of ‘Getting Involved in Research’ in the analysis strengthened the project findings given their in-depth knowledge of the course while also providing a forum to democratise power-sharing. Given that the PTE training session was deliberately ‘light-touch’ we also deemed this to be a strength, given that the purpose of the analysis was not to professionalise the skills of those with lived experience or those from the community and voluntary sector but rather gain insight from their background experience. Considering that there was consensus between the co-researchers and the independent academic researcher (78% similarity), this suggests that there is validity in the method of PTE while also being aware that interpretation in qualitative findings generally has a certain subjectivity.

Although the method of using Miro was necessitated by the restrictions (Covid-19) using the method face to face may have generated more interactivity in discussions with the co-researchers. Though the number of quotes (n = 94) was found to be sufficient to ensure the process was manageable in the given time period, this is a relatively small number of quotes relative to the entire data set. This selection process may therefore be a notable limitation of the approach and create a potential source of bias. To further minimise this bias, we also ensured that the independent academic researcher read the entire data set and based groupings of the selected quotes on this knowledge. The group was intentionally mixed with co-researchers coming from academic backgrounds, lived experience, community and voluntary sector and the DoH and caution was applied to ensure that all co-researchers had an opportunity to voice their perspectives. However, it may have been more effective to have had a group solely with representation from those with lived experience who were participants in ‘Getting Involved in Research’.

## Conclusion

Overall, given that there is very little existing knowledge about the impact of introductory research courses for those who have little or no experience of conducting research; ‘Getting Involved in Research’ has contributed uniquely and innovatively to the evidence base for how to engage with and motivate those who have experience of health and social care to become actively involved in research. Participants in ‘Getting Involved in Research’ reported that the training contents were applicable, relevant, fostered awareness of research methods and anticipated that it would support their involvement in research. Furthermore, the majority of the course participants indicated that a participatory approach was evident and contributed positively to the learning environment. This study also correlates with existing studies that confirm PTE as a reliable method of thematically analysing data while also effective in engaging co-researchers meaningfully in research [23, 36, 39–41—Authors' own]. This study demonstrates that ‘Getting Involved in Research’ may be helpful to train those with lived experience and their care partners however, further research following up on the application of the course learning would be required to ascertain effectiveness. Given the success of ‘Getting Involved in Research’, there is an urgent need for funders to fund training and support for lived experience contributors and other stakeholders in research across conditions and populations as facilitating this exchange has evidently supported mutual learning and has enhanced the impact on participants [11–16, 18, 30].

### Future directions

Future research should investigate the impact of courses such as ‘Getting Involved in Research’ has on those who have been involved as stakeholders in the design process as is also important to more fully understand the impact that this exchange has had on the course development members. As Staley et al., [54] state “Researchers learn from an exchange of knowledge with patients/carers, which influences their plans and actions.” ‘Getting Involved in Research’ may also be enhanced by a more individualised approach with greater consciousness of levels and abilities if future groups remain diverse in their levels of skills and experience. The social aspects of the course could also be increased to include more interactive discussions while reducing the density of course content to include more informal group work. Future research might also explore the implementation of other process that could further devolve power and enable meaningful lived experience contribution and more leadership in course design and delivery. A follow-up course and opportunities to apply research skills in practice may also be beneficial to further develop participants’ confidence in using the skills gained through ‘Getting Involved in Research’.

## Supplementary Information

**Additional file 1**. Participant quotes.

**Additional file 2**. Validation of thematic groupings of quotes.

## Data Availability

All data generated are included in this article [and its supplementary files].

## Abbreviations

PPI: Patient and public involvement
PTE: Participatory theme elicitation
DoH: Department of Health
MHF: Mental Health Foundation
QUB: Queen’s University Belfast
UU: Ulster University
DRILL: Disability Research on Independent Living and Learning

## Footnotes

1 In this project the focus was on people with their own experiences of using health and social care, however the language in the area of research referring to those with lived experience is still developing. In this article, the term ‘lived experience’ will be used from this point onwards to refer to individuals with experiences of health and social care.
