# Supplementary material for: ‘Getting involved in research’: a co-created, co-delivered and co-analysed course for those with lived experience of health and social care services
Source: Res Involv Engagem. 2022 May 16;8:20. doi: 10.1186/s40900-022-00353-x (PMC9109673; doi:10.1186/s40900-022-00353-x)
Supplement: Supplementary file 1 — Additional file 1. Participant quotes. [file 40900_2022_353_MOESM1_ESM.docx]

**Additional File 1:** Participant Quotes

| PQ 1. The course presenters made it a very friendly and interesting course and also a lot of the learning came from the sharing from the other delegates. |
| --- |
| PQ 2. A balance between theory and practice (applied research); A balance between delivery and participant involvement; An opportunity to apply learning through an assessed assignment (voluntary) |
| PQ 3. I really enjoyed the course. The lecturers imparted their passion for research & kept everyone engaged. I also feel the group dynamic was excellent. Everyone engaged & were enthusiastic about the course. |
| PQ 4.I really enjoyed interacting with other participants and hearing other people's stories. I liked having variety of speakers and different topics we touched and discussed. |
| PQ 5. Zoom made it easily accessible, but I think it could be much more powerful face-to-face (or 50/50). |
| PQ 6. Recognise these [reflective journals] are very important, at first thought excessive but soon realised that they are a valuable resource. |
| PQ 7. I really thoroughly enjoyed the whole experience from the knowledge gained to tutors and other participants. The tutors wealth of knowledge and insight of how we were feeling at different points was also spot on. |
| PQ 8. I said agree as I partly didn't know what exactly to expect. I expected a well organised, knowledge based learning platform to both further my own personal goals and also professional goals and that's exactly what I got |
| PQ 9. I think it would be nice to meet more of the participants- in our first breakout session we introduced ourselves but didn't get the opportunity to do this for the remainder of the week. I think taking some time early in the week to explain your situation to everyone would be useful. |
| PQ 10. The only limitation I experienced was with the virtual element. At times when there were technical issues it was difficult, however if it wasn't for the course being virtual due to my work & caring commitments, I'm unsure if I would have been able to commit |
| PQ 11. I feel the content was pitched at the right level. It was interesting, kept me engaged & made me feel excited about the prospect of getting involved with research |
| PQ 12. [I enjoyed the] real world focus. Involvement (peer and practitioner) as well as research. Participant engagement. Rich learning. |
| PQ 13. I would say it was higher than my expectations… Commitment to increasing peer involvement feels real and all contributions were valued/valuable. |
| PQ 14. Approach felt collegial/collaborative… Good to have people presenting from perspective of lived experience and research involvement. |
| PQ 15. Valuable learning from course participants was shared/received with interest, not defensively... I also much appreciated the recognition of what interactions 'feel' like and the importance of attention to detail and sensitivity of power dynamics, including who controls which room we are in :). |
| PQ 16.Interesting to hear more 'radical' perspective around and other important questions posed about current norms. Thank you for bringing lens to quality assurance - made me more think about issues of accessibility and why it is valuable to have new perspectives. |
| PQ 17. My confidence and interest in becoming more actively involved in research has grown over this week. Though we have covered a lot of ground, the structure has felt manageable and I could sense the thought and care that has gone into this course. |
| PQ 18. The practical application of what we were learning. Seeing how I as a service user could become involved and how my opinion input does matter. I learnt a lot from others in breakout rooms. |
| PQ 19. I felt rather inadequate and awkward in some of the breakout rooms. The gap between my level of knowledge as a service user and someone who is already involved in research was possibly too large a gap to be able to incorporate both levels in the same course. |
| PQ 20. I learned a lot in a pleasant environment. I liked that so many of us contributed. Presenters were relaxed, pleasant, encouraging, knowledgeable, and professional. |
| PQ 21. I was expecting it to be more relating to nursing and didn't realise it was a course for everyone but I still thoroughly enjoyed it and enjoyed mixing with people from different backgrounds. |
| PQ 22. [The strengths of the course were]The friendliness, course content, good discussions, opportunities to reciprocate dialogue between tutors and participants.and the informality of the overall experience. |
| PQ 23. The diversity of the participants was the greatest strength. |
| PQ 24. I really enjoyed participating in the course and the interactions with other attendees, despite this being on Zoom...which does make it much harder for engagement with others. |
| PQ 25. The attendees were also understanding and welcoming. This in it self helps you to feel comfortable…Peer support through learning is incredibly valuable. I thank all those that contributed. Discussion based activities are great. |
| PQ 26. The different levels of understanding did mean that at times I felt my knowledge was so limited therefore sat back and listened quietly. Discussion based activities are great. |
| PQ 27. All the things that I have learned this week I will be incorporated into our services. I will be sharing with colleagues and adding to our overall evaluation framework. |
| PQ 28. Well, it is on Zoom. Sitting at a PC all day is tiring. You miss the one to one/face to face interaction that you normally get if you were in the same room. Also I had background noise and general distractions at home, so sometimes not easy to focus. But can't be helped!! |
| PQ 29. Wasnt sure what to expect from course or was it for me but it was very clear what it was about and was left enough information to take if further like training etc |
| PQ 30. Well organised, good timing finished when it said. Good discussions and time to talk to lecturers if needed to over breaks. Good overall balance of course |
| PQ 31. The course was really well thought through. Variety of speakers and topics were very engaging. It seemed very professional. Also thank you to those who shared their personal stories. |
| PQ 32. I felt comfortable and not under pressure. A good environment to learn in!!  Thank you, thank you :) |
| PQ 33. Not too demanding. There was breakout rooms and encouragement to put camera on but not too much to the point it was off putting or overwhelming. |
| PQ 34. I feel that an effort was made for everyone to be able to participate, especially with the Zoom check at the beginning. It felt accessible and I felt welcomed in the environment. |
| PQ 35. Maybe some activities/strategies on how service users can be involved in research - what does this look like in a real example, in different situations. The course showed how important this was, and how service users can be part of the research team (co-production), but what does this look like? |
| PQ 36. I also think it would be better to have more than 2 examples of Service Users |
| PQ 37. Zoom is great so you don’t have to travel but I guess speaking to other classmates face to face is always better |
| PQ 38. All the tutors were excellent, they really engaged with participants and included and encouraged everyone - their facilitation skills (and knowledge) ensured the course was successful, in my opinion. Thank- |
| PQ 39. I will find the course useful and the facilitators were very keen to gauge the different backgrounds and needs of participants to ensure the course is flexible to the needs of the participants. |
| PQ 40. I have learnt a lot from the course and it has raised my level of confidence about research and would definitely recommend it to others. |
| PQ 41. I also noticed other people letting the group see more of them and what they are interested in and already know and hope to learn. It helps a lot to feel that. |
| PQ 42. service user involvement history is one of my pet projects in understanding and valuing the voice and expertise of the service user ( carer and professional with lived experience) - I really enjoyed learning about this. it was presented in an encouraging and focussed way that invited involvement and further study. |
| PQ 43. Perhaps some condensing of the course material might help in the overall timing, giving more time to ponder and reflect when thinking about answers and responses. |
| PQ 44. At the beginning I found myself interacting within small breakout rooms but not feeding back in the bigger group. By Wednesday I felt very comfortable interacting with my group and volunteered to feed back to the bigger group |
| PQ 45. I didn't think it would be quite so intensive and as Im retired and take my time over things I will need to crank up my learning skills. |
| PQ 46. Too much interruption through participants speaking. I feel it would be better to focus on the presentation and use the chat for comments. |
| PQ 47 Thanks to the team involved for an educational, informative, enjoyable week. With my experience of organizing meetings in the past and organising and hosting Zoom sessions currently ... The emails every morning I am sure avoided a few requests for the links even though you had already supplied them! |
| PQ 48. A teaching expert might have a different opinion about teaching techniques. I am happy that a variety of Zoom facilities were used in an appropriate manner and effectively. |
| PQ 49. After the first breakout session there was a comment made by one of the speakers, asking if people will volunteer to share their discussions or should they be randomly called out by names. I didn't think that was very nice because not everybody feels comfortable speaking in a large group. |
| PQ 50. '-Quicker confirmation on securing the course placement -Shorter lunch break -Less breakout sessions, more general discussion in a large group -One survey at the end of afternoon session that would summarize both morning and afternoon session that day. |
| PQ 51. It is people with lived experience that can really make a difference, I began three and a half years ago as a ppi member and trained on their 'expert patient' course this was the beginning of getting involved in many initiatives, this was co-production in action |
| PQ 52. Even though the aim was slightly low for me, I was still happy because it gave me a lot of confidence knowing that I can understand everything that was said today. |
| PQ 53. potential amendment...Maybe a half day pre-course lecture for those who have no experience just to explain the premise of the course and some of the terms used in the course. Also maybe explaining some of the methods of teaching, e.g breakout rooms( the first two nearly triggered a panic attack). |
| PQ 54. The tutors did help to calm my initial anxiety and helped me to see this is possibly something I can pursue.. I was in a lovely breakout room but some other participants and their vast experience and knowledge made me wonder am I on the wrong course! |
| PQ 55. I enjoyed relaxed approach, seeing the slides and having the opportunity to meet participants in the break out room. Glad we were able to explore idea of Peer-Led Research as part of the research 'context'. Overall good opportunity for understanding key ideas and some critical reflection. |
| PQ 56. A small point- I know this can be challenging given the format and facilities on zoom, but it could help the entire group if we were aware of the different experience and confidence levels (of other participants) in the field of research |
| PQ 57. Enjoyed the discussion in breakout room. But greatly helped that I was with some very experienced people. |
| PQ 58. Approach felt engaging and human, relating to real world and presenters were responsive. Pitch felt right, not too overwhelming and up-to-date (when the discussion on Peer-Led research was included |
| PQ 59. I much appreciate the thoughtful content and awareness of what and how we communicate |
| PQ 60. Good to discuss in small groups. I have gained new knowledge, awareness and had interesting open discussions. |
| PQ 61. I'm judging confidence through my level of participation - and I have felt confident to do so - even to initiate group activity because by doing so I can then get others to convey their views, from which I and the others can benefit |
| PQ 62. I think the tutors pitched it right coming from someone with no depth of knowledge in research. They adapted when necessary to be inclusive which was great. Enthusiastic engaging and knowledgeable |
| PQ 63. Course tutors were very aware of the diverse audience and were mindful of this which was great. |
| PQ 64. There was a very relaxed atmosphere and the group discussion worked well- combination of chat feature and people giving their views was well-managed. |
| PQ 65.The importance of peer involvement came across strongly & the benefits were highlighted. I was particularly interested in the discussion around tokenism. It has highlighted that some work projects that I have been asked to be involved in were exactly this. I feel after today's lecture I am in a position to question/challenge in future & hopefully move towards changing mindset |
| PQ 66. I am a hands-on learner & am hopeful the assignment will provide this opportunity in order to help increase my confidence |
| PQ 67. Very relaxed atmosphere with practical examples, also very useful chat examples from participants. |
| PQ 68. Lots of imparted examples from attendees and good breakout discussions - helps to enrichen what is presented |
| PQ 69. I will be more aware of not just how 'a message is articulated' but also to 'how it is disseminated' - no point in having a well-articulated message if it is ineffectively communicated! |
| PQ 70. I feel dissemination of actual real opportunities was brilliant as sometimes it's a bit overwhelming in where to even start. Lots of useful websites/ resources but also the offer of support from tutors was great |
| PQ 71. I feel there was a huge amount of information covered in a short space which the tutors were also very aware of which was great. It's a difficult balance, for me I think it was spot on |
| PQ 72. Breakout rooms are really useful to get wide variety of experience and opinions and see things from different objectives. |
| PQ 73. I especially appreciate in this course that presenters include learning from mistakes and acknowledge that there are many influences, including unconscious bias. |
| PQ 74. As the week has evolved, the learning feels more like a shared endeavour. |
| PQ 75. Was lovely to have the course delivered by familiar faces throughout the week without the monotony of one tutor. Multiple presenters I felt was a great approach especially on zoom to allow the management of chat etc. but also to hear different perspectives on same subject areas |
| PQ 76. I feel increased confidence in bringing questions about involving people with lived experience to the larger group. |
| PQ 77. Glad course dedicated time to providing information, support and encouragement to explore/create opportunities and hear about this from a personal perspective. I much appreciate the thought and care that has gone into PowerPoint/media content and personal sharing. |
| PQ 78. How could one not benefit from this? It was for everyone even if everything did not apply to all, there was something there for everyone to take away and apply. |
| PQ 79. Yes you felt like it was possible to conduct research, it wasn't just something academics did. You felt valued for your life skills and not qualifications |
| PQ 80. The tutors made me feel included and I loved that the tutor wanted to know our future goals in 6 months |
| PQ 81. I know only I can shape my research journey but I'm not sure I have enough confidence to take the next step. I am hopeful the assignment will help to give me an indication if it is something I am capable of |
| PQ 82. A worked example (if possible) could be shown during the sessions, e.g. short extracts from "real" quality appraisal documents to show what points were considered, short extracts from a research proposal that would be considered of low quality (and why) as well as high quality research) and why? What does it actually look like in "real" terms? |
| PQ 83. [I'd recommend] More interactive stuff! The role play, the quizzes, the breakout rooms - they work so well! |
| PQ 84. All the tutors have been very interactive this week and have created a very relaxed atmosphere within the group. This has added to the learning when hearing about lots of personal perspectives on taking part in research and how research was disseminated. |
| PQ 85. Very glad to have taken part in the course and shared this rich learning experience with everyone's contributions. Feels like an opening for new possibilities that are meaningful, however long this takes. |
| PQ 86. I learnt from other participants from real experiences which was great. The only negative to that in using past experiences does perhaps not allow the same investigation for those with no experience to make it applicable. However I think the benefit of those with experience of research does outweigh the negative |
| PQ 87. The content is brilliant, but the allocated time made it difficult to go into the topic more deeply. |
| PQ 88. Beginning to find break out discussion groups very helpful and help build confidence. |
| PQ 89. I much appreciated sharing of personal perspectives. Sometimes I find it helpful (more equalising) when we also value aspects of our lives sensitively out with our service status and roles when we say who we are. |
| PQ 90. Everything was clear and understandable. Could relate to Fiona and felt tutors were speaking a language I understood. Can definitely see how information could be applied. |
| PQ 91. Really good how we got time to interact with each other in groups, this is so important for those who are less inclined to talk in larger groups |
| PQ 92. 'lived experience' experience is vital if we are to be any good at involving lived experience in designing and delivering the outcomes and outputs that best suit the citizens who are supposed to be helped by the service and community being developed and served. |
| PQ 93. It was great to hear everyone's knowledge and hear how combined we know it all! Lol! Which I think was the biggest message of the session; form a team of people with all the skills needed, combined. It also illustrates how valuable lived experience provides the answers the facilitators could have talked at us instead. |
| PQ 94. I loved how our opinions and discoveries and half formed thoughts were taken on board and valued - even to the extent of the facilitators asking themselves 'are they teaching too formal a way to do this service user involved research?" |
