# Supplementary material for: ‘Getting involved in research’: a co-created, co-delivered and co-analysed course for those with lived experience of health and social care services
Source: Res Involv Engagem. 2022 May 16;8:20. doi: 10.1186/s40900-022-00353-x (PMC9109673; doi:10.1186/s40900-022-00353-x)
Supplement: Supplementary file 2 — Additional file 2. Validation of thematic groupings of quotes. [file 40900_2022_353_MOESM2_ESM.docx]

**Additional File 2:** Validation of thematic groupings of quotes

| **Theme based on co-researcher group**  **agreement** | **Quote IDs from co-researchers**  **Agreement** | **Quote IDs from independent academic researcher** | **Percentage of quotes in same theme based on comparison with an independent academic researcher** |
| --- | --- | --- | --- |
| 1. Participatory Approach | PQ4, PQ12, PQ13, PQ14, PQ15, PQ18, PQ23, PQ25, PQ41, PQ42, PQ51, PQ65, PQ67, PQ68, PQ74, PQ79, PQ85, PQ86, PQ89, PQ92, PQ93 | PQ4, PQ12, PQ13, PQ14, PQ15, PQ18, PQ25, PQ35, PQ42, PQ51, PQ52, PQ65, PQ68, PQ74, PQ79, PQ85, PQ86, PQ89, PQ92, PQ93 | 90% |
| 2. Confidence | PQ17, PQ40, PQ44, PQ52, PQ61, PQ66, PQ76, PQ81. | PQ26, PQ40, PQ41, PQ44, PQ61, PQ66, PQ76, PQ81, | 75% |
| 3. Format | PQ5, PQ9, PQ10, PQ24, PQ28, PQ33, PQ37, PQ46, PQ47, PQ48, PQ49, PQ57, PQ60, PQ72, PQ83, PQ88, PQ91. | PQ5, PQ9, PQ10, PQ24, PQ28, PQ37, PQ46, PQ47, PQ48, PQ49, PQ50, PQ57, PQ60, PQ72, PQ83, PQ88, PQ91. | 94% |
| 4. Learning Environment | PQ1, PQ3, PQ7, PQ20, PQ22, PQ32, PQ34, PQ38, PQ39, PQ54, PQ55, PQ58, PQ62, PQ63, PQ64, PQ75, PQ78, PQ80, PQ84, PQ94. | PQ1, PQ3, PQ7, PQ32, PQ33, PQ34, PQ38, PQ39, PQ54, PQ55, PQ58, PQ62, PQ64, PQ67 PQ75, PQ78, PQ80, PQ84, PQ94 | 85% |
| 5. Future Developments | PQ2, PQ21, PQ35, PQ36, PQ43, PQ50, PQ53, PQ69, PQ82. | PQ2, PQ21, PQ22, PQ23, PQ51, PQ56, PQ63, PQ69 | 33% |
| 6. Course Content | PQ6, PQ8, PQ11, PQ16, PQ19, PQ26, PQ27, PQ29, PQ30, PQ31, PQ45, PQ56, PQ59, PQ70, PQ71, PQ73, PQ77, PQ87, PQ90. | PQ6, PQ8, PQ11, PQ16, PQ17, PQ19, PQ20, PQ27, PQ29, PQ30, PQ31, PQ36, PQ43, PQ45, PQ53, PQ59, PQ70, PQ71, PQ73, PQ77, PQ82, PQ87, PQ90 | 89% |
| **Overall % Agreement with co-researchers grouping of quotes** | | | 78% |
